# Integrating narrative medicine into post-genocide Rwandan healthcare: a call for trauma-responsive medical education

**DOI:** 10.3389/fmed.2026.1859152

**Published:** 2026-09-22

**Authors:** John Mugisha, Julius Niyibizi, Isimbi Badaga Diane, Emmanuel Nizeyimana, Claudia Buhendwa, Yang Xiaolin

**Affiliations:** 1 Rwanda Implementation Science & Health Systems Lab (RISHS Lab), Kigali, Rwanda; 2 Southern Medical University, Guangzhou, China; 3 Narrative Medicine Center, Southern Medical University, Guangzhou, China

**Keywords:** health professions education, narrative competence, narrative medicine, reflective practice, Rwanda, social competencies, trauma-responsive communication

## Abstract

This narrative review examines the potential integration of narrative medicine into Rwanda's post-genocide healthcare system, where long-term psychological consequences continue to shape patient experiences and clinical practice. Despite significant improvements in health system performance since 1994, Rwanda's post-genocide context continues to shape emotionally demanding clinical encounters for patients and healthcare professionals. Narrative medicine refers to the clinical and educational capacity to attend to, interpret, and respond ethically to patients' illness stories. International literature suggests that narrative-based education may strengthen social and relational competencies, including empathy, perspective-taking, reflective capacity, and communication; however, these findings are context-dependent and require Rwanda-specific evaluation. In this review, the Chinese clinical narrative thinking framework serves as one possible analytic scaffold, not a model to be transferred uncritically. Its value is that it organizes narrative competence into four teachable clinical domains: narrative diagnostic thinking, narrative decision-making, narrative patient education, and narrative care or intervention. These domains are considered alongside foundational narrative medicine, illness narrative, reflective practice, and trauma-informed care frameworks. Although Rwanda has strong traditions of storytelling, testimony, and collective healing, these community and public practices are not direct equivalents of clinical narrative medicine. Narrative medicine has not yet been systematically integrated into Rwandan medical education or routine clinical practice, and any future integration would require trauma-sensitive safeguards, careful professional boundaries, and empirical evaluation. This review integrates deliberately selected global and context-specific literature to examine the theoretical underpinnings of narrative medicine, its significance to Rwanda's sociocultural storytelling and collective healing, and the gaps in medical education. While Rwanda-specific empirical evidence remains limited, the convergence of ongoing mental health needs, national workforce expansion, and strong narrative traditions presents a timely opportunity to develop trauma-responsive healthcare models relevant to post-conflict societies globally.

## Introduction

Rwanda's recovery following the 1994 genocide against the Tutsi represents one of the most remarkable public health transformations of the modern era (1, 2). In the decades since, the country has rebuilt a devastated healthcare system, expanded community-based health insurance, strengthened maternal and child health services, and achieved substantial improvements in population health indicators (2–4). Life expectancy at birth in Rwanda rose from 28 years in 1994 to around 69 years, signaling a strong and resilient health system (1, 3).

This review specifically examines whether concepts of narrative medicine could be responsibly adapted to Rwandan health professions education. It is primarily focused on undergraduate and postgraduate training, faculty development, clinician reflection, trauma-responsive communication, and safeguarding. We consider clinical, sociocultural, and psychosocial evidence only in relation to how it can inform educational design; narrative medicine is not presented as an established clinical intervention in Rwanda.

However, behind all these positive aspects, Rwanda still faces a complex psychological landscape formed by experiences of trauma, loss, and intergenerational memories. Survivors still have post-traumatic stress disorder (PTSD), depression, and anxiety years after the genocide against the Tutsi (5). These psychological consequences extend beyond survivors to their families and communities. Healthcare professionals may also encounter diverse trauma-related narratives in clinical settings, each shaped by an individual's experiences, relationships, silences, and meanings (5, 6).

Health determinants are often shaped by a combination of biological, behavioral, and social factors (7–9). Social connectedness and meaning-making processes (storytelling and shared narratives) can be significant in psychological recovery and resilience in post-conflict environments (10). Such strategies can alleviate isolation and social disintegration after collective trauma by facilitating interpersonal relationships through active listening and sharing lived experiences. Such a view is substantiated by the findings of the so-called Roseto effect, in which high social cohesion among a community of Pennsylvania Italian immigrants was linked with a longer lifespan despite low biomedical benefits (11).

There are specific physical and emotional struggles that healthcare providers (HCPs) face. Having many patients, limited resources, and seeing them suffer can contribute to experiencing emotional exhaustion, moral distress, and secondary traumatic stress (12). In these situations, HCPs may experience negative impacts on professional identity, provider-patient relationships, and treatment quality, contributing to burnout and worsening patients' conditions. Introspective and empathic strategies, together with building resilience, are crucial in such situations, as they may allow HCPs to cope with stress more effectively and establish better connections with their patients (13). The concept of narrative medicine provides a specific approach toward achieving this goal through developing clinical narrative thinking, a reasoning process complementary to evidence-based thinking (14, 15). While evidence-based reasoning emphasizes objectivity, consistency, and empirical validation, narrative thinking aims to understand the unique context of a patient's illness story, since a person's experience can be considered diagnostic information in itself (16).

This review uses several key terms in defined ways. Narrative medicine refers to clinical and educational practices that strengthen clinicians' capacity to listen to, interpret, and respond ethically to patients' illness stories. Narrative competence is the ability to recognize meaning, emotion, silence, uncertainty, and context within clinical encounters. Clinical narrative thinking applies this competence to diagnosis, decision-making, patient education, and care. Narrative therapy is distinct: a psychotherapeutic approach delivered by trained mental health professionals that helps individuals reframe problem-dominated life stories. Trauma-responsive care recognizes the effects of trauma, protects dignity and choice, avoids re-traumatization, and connects distressed individuals with appropriate support. Narrative connection refers to the trust that can emerge through attentive listening, recognition, and respectful interpretation.

Narrative competence also intersects with social and emotional competencies in health professions education. Attentive listening, perspective-taking, reflective self-awareness, recognition of emotion and meaning, and empathic communication are relevant to both narrative practice and effective interpersonal care. Narrative competence is not, however, synonymous with emotional intelligence. Emotional intelligence broadly concerns the ability to perceive, understand, and regulate emotions in oneself and others, whereas narrative competence focuses on how clinicians attend to, interpret, and respond ethically to patients' stories, meanings, and contexts (17, 18). Narrative medicine may therefore offer an educational approach to practicing selected social and relational competencies without being treated as emotional-intelligence training itself.

## Methods

### Design and rationale

This article is a narrative review and conceptual synthesis. A narrative review design was selected because the question is exploratory, context-sensitive, and not yet supported by a substantial body of Rwanda-specific empirical research (19). The aim was not to estimate the intervention's effectiveness or produce a pooled summary of outcomes. Rather, the review examined how global literature on narrative medicine, reflective practice, clinician well-being, illness narratives, and trauma-informed care might inform future health professions education in post-genocide Rwanda. A narrative review is therefore used as a preliminary step to map the conceptual terrain and identify testable questions, rather than substituting for the original, Rwanda-based research this field ultimately requires.

### Search strategy and source identification

Sources were identified through purposive searches of PubMed, Scopus, Google Scholar, and university library databases up to January 2026. Search terms included combinations of “narrative medicine,” “narrative competence,” “clinical narrative thinking,” “reflective writing,” “illness narratives,” “medical education,” “patient-provider communication,” “burnout,” “trauma-informed care,” “post-conflict health,” “Rwanda,” “genocide,” “sociotherapy,” “Gacaca,” “narrative therapy,” “trust,” “provider identity,” “patient-provider concordance,” and “trauma disclosure.” We also reviewed reference lists of relevant articles and books to identify foundational, theoretical, and Rwanda-specific sources.

Alongside peer-reviewed databases, we examined publicly accessible grey literature, including Ministry of Health publications, medical school curriculum blueprints, and relevant policy documents. We found no rigorous assessments or published analyses of the curriculum explicitly addressing narrative medicine, reflective writing, or clinical narrative thinking in Rwandan medical schools.

### Inclusion and exclusion criteria

We included peer-reviewed empirical studies, theoretical articles, books, book chapters, and relevant reports that contributed to at least one of the following themes: narrative medicine theory; clinical narrative thinking; reflective practice in medical education; clinician empathy and well-being; patient-provider communication; trauma-informed care; Rwandan post-genocide psychosocial healing; sociotherapy; narrative therapy; and community-based or testimonial practices in Rwanda. We excluded duplicate sources, inaccessible full texts, materials unrelated to the review question, non-academic sources without clear conceptual or empirical value, and sources whose claims could not be clearly linked to the manuscript's focus.

### Source selection and synthesis

Because this was a narrative review, we selected sources purposively and interpretively rather than systematically or exhaustively. We did not conduct a PRISMA-style systematic review, formal risk-of-bias assessment, meta-analysis, or quantitative evidence grading. The authors read and discussed sources, and organized the synthesis around recurring themes: narrative medicine theory, clinical narrative thinking, Rwanda's sociocultural narrative practices, medical education gaps, clinician well-being, ethical safeguards, and future research priorities. Greater interpretive weight was given to foundational narrative medicine literature, peer-reviewed empirical studies, and Rwanda-specific psychosocial literature.

### Methodological boundaries

The review deliberately distinguishes established evidence from contextually plausible extrapolation. Evidence that narrative medicine may improve empathy, reflection, communication, or clinician well-being comes mainly from settings outside Rwanda. Rwanda-specific evidence is stronger for sociotherapy, testimony, reconciliation-related narrative practices, and narrative therapy than for narrative medicine itself. Accordingly, the manuscript treats integrating narrative medicine into Rwandan medical education as a hypothesis requiring co-design, feasibility testing, trauma-safety evaluation, and empirical study, rather than a proven intervention.

### Ethical considerations

This article is based on previously published literature and did not involve human participants, primary data collection, or access to identifiable personal information. Formal ethics approval was therefore not required.

## Thematic findings

### Theoretical foundations for a medical education application

Narrative medicine has evolved into an interdisciplinary field, emphasizing the role of narrative competency, as defined by Rita Charon, as a clinical practice approach that relies on narrative competence, which is the capacity to identify, interpret, and be affected by narratives of illness (20). This methodology draws on phenomenology, narrative identity theory, and critical social theory, asserting that narrative competence is essential for effective clinical practice (21). It incorporates reflective writing, close reading of literary and clinical texts, and attentive listening techniques into healthcare training and provision (20, 22, 23).

One framework that may help translate narrative medicine into teachable clinical skills is the clinical narrative thinking framework developed in Chinese narrative medicine scholarship. We do not present this framework as Chinese in the sense that Western or global narrative medicine lacks narrative attention. Foundational narrative medicine already emphasizes close listening, interpretation, ethical attention, and reflective practice. The distinctive usefulness of the clinical narrative thinking framework is pragmatic: it organizes narrative competence into four clinical reasoning domains: diagnosis, decision-making, patient education, and care or intervention that can be mapped onto medical education and routine clinical work. For Rwanda, the framework serves as an analytic scaffold rather than an imported model. Local co-design, faculty preparation, and empirical evaluation must test its relevance.

The clinical narrative thinking framework builds on, rather than replaces, established narrative medicine scholarship. Charon's concept of narrative competence, the capacity to absorb, interpret, and be moved by patients' stories, provides the ethical and relational foundation for all four domains described below (20). Kleinman's classic distinction between disease, as biological dysfunction, and illness, as the patient's lived and meaning-laden experience, is particularly relevant to narrative diagnostic thinking, since it explains why attention to a patient's own account can carry diagnostic and therapeutic value that laboratory findings alone do not capture (24). Frank describes restitution, chaos, and quest as interpretive narrative types, not necessarily phases in a linear or circular progression. Restitution narratives anticipate recovery and return to previous health. Chaos narratives convey suffering that resists temporal order, explanation, or a realistic prospect of recovery. Quest narratives acknowledge suffering while seeking meaning, testimony, or purpose. Individuals may move between or combine these narrative forms (25). Therefore, the clinical narrative thinking framework is organizational rather than conceptually novel: it translates established narrative medicine ideas into four domains suited to clinical teaching, making it a practical, testable scaffold for Rwanda rather than a competing theoretical import.

Biomedical diagnosis concentrates on laboratory results and imaging findings. In contrast, narrative diagnosis involves extracting and analyzing the patient's illness narrative within its comprehensive context, including temporal sequence, causal attributions, emotional responses, and social implications. This approach may be relevant in trauma-exposed contexts, where patients sometimes present with medically unexplained symptoms or where physical symptoms are entangled with psychological and social suffering (26, 27). Nonetheless, narrative diagnostic thinking should be regarded as an adjunct to biomedical assessment, not a replacement. Its potential value in Rwanda is a hypothesis that requires careful evaluation.

Second, narrative decision-making facilitates shared decision-making by acknowledging that both the patient and the clinician bring their story to the consultation. Decision-making involves more than calculation; it includes values, risks, and stories of future possibilities. Narrative decision-making supports informed consent and treatment adherence by clarifying these frameworks; it helps patients understand the risks and benefits of different treatment options in a more relatable context.

Third, narrative patient education changes how physicians communicate health information to patients. Unlike traditional approaches that share information without context, narrative patient education uses storytelling and analogy to help patients understand illnesses and possible treatment plans. This approach is particularly useful in cross-cultural scenarios where biological perspectives do not align with local explanations of disease, because it helps patients understand their health concerns within their cultural context.

Fourth, narrative care and intervention thinking involves sustained attention to patients' changing illness experiences throughout care. In ordinary clinical practice, such care may include reflective practice, narrative-informed follow-up, and communication support; psychotherapy should remain within the scope of appropriately trained mental health professionals. These four dimensions operate in dialectical complementarity to evidence-based thinking. While evidence-based thinking emphasizes objectivity, regularity, and empirical verification, which are essential for safety and effectiveness in interventions, narrative thinking highlights the uniqueness, situational aspects, and relational aspects of care. Neither mode is sufficient alone; effective clinical practice requires integrating both.

Cultivating narrative competence may support more attentive and ethically responsive relationships between healthcare professionals and patients (20, 22, 23). This relational aim may be particularly relevant in post-genocide settings, where trust can be shaped by unequal and ethically distinct histories of victimization, survival, perpetration, complicity, rescue, displacement, and intergenerational family memory (5, 10, 28, 29). These experiences should not be collapsed into a generic dispute. Survivors' experiences of violence, bereavement, and loss of safety are not equivalent to the guilt, shame, fear, social consequences, or inherited burdens that may be experienced by perpetrators, their relatives, or other community members (5, 28, 29). Narrative medicine cannot reconcile these histories or substitute for psychotherapy (20, 22, 30). Its more limited role is to prepare clinicians to listen without equating different experiences, avoid imposing redemptive interpretations, protect patients from pressured disclosure, and recognize when specialized mental health or reconciliation support is required (20, 22, 31, 32).

Global evidence suggests that narrative-based educational activities can support empathy, perspective-taking, reflective capacity, communication, and professional identity formation among health professions learners (33, 34), including capacities relevant to social and relational competence. Evidence on clinician burnout and resilience is promising but uneven; some studies report improvements after narrative medicine or reflective-writing workshops, while broader conclusions remain limited by small sample sizes, heterogeneous interventions, and concentration of studies in high-income settings (35, 36). For Rwanda, these findings should therefore be interpreted as a rationale for feasibility and acceptability studies, not as proof of effectiveness. Before making strong claims about patient trust, diagnostic quality, PTSD symptoms, or health outcomes, direct Rwanda-specific research is necessary.

Foundational work in narrative medicine and illness narratives emphasizes that clinical care requires attention not only to disease but also to the patient's lived experience of illness, meaning, suffering, and social context. To clarify the conceptual and practical distinctions between narrative medicine and narrative therapy, Table 1 summarizes their differences in aims, practitioners, settings, and Rwanda-specific evidence.

**Table 1:** Key distinctions between narrative medicine and narrative therapy for trauma-responsive implementation in Rwanda.

Feature	Narrative medicine	Narrative therapy
Primary aim	Develop clinicians' narrative competence, reflective capacity, ethical listening, communication, and patient-provider relationships.	Provide psychotherapy that helps individuals or groups re-author problem-saturated life stories and work therapeutically with distress, identity, and trauma.
Primary professional role	Physicians, nurses, medical students, educators, and other healthcare providers who use narrative skills in routine care or education.	Trained mental health professionals, including counselors, psychologists, psychotherapists, or clinicians with specific psychotherapy training and supervision.
Training requirements	Training in attentive listening, close reading, reflective writing, trauma-responsive communication, professional boundaries, confidentiality, and referral recognition.	Formal psychotherapy training, supervision, trauma-management skills, risk assessment, and competence in therapeutic intervention.
Usual setting	Medical education, clinical consultations, ward rounds, reflective groups, faculty development, and clinician well-being activities.	Structured therapy sessions, mental health clinics, psychosocial programs, or supervised community mental health interventions.
Intervention type	Educational and relational practice supports listening, interpretation, reflection, shared decision-making, and communication.	Psychotherapeutic intervention may involve structured exploration of traumatic memories, identity, family relations, and meaning-making.
Expected outcomes	Expected outcomes include potential improvements in empathy, reflection, communication, professional identity formation, and clinician well-being; however, the effectiveness specific to Rwanda remains untested.	Potential reduction in psychological distress, anxiety, depression, or PTSD symptoms when delivered appropriately; evidence depends on population and intervention design.
Ethical risks	Narrative pressure, confidentiality breaches, emotional overload in learners or patients, inappropriate interpretation of stories, or blurring boundaries with psychotherapy.	Re-traumatization, inadequate containment of distress, dependency, poor supervision, or therapy delivered by insufficiently trained providers.
Rwanda-specific evidence	No direct empirical studies of narrative medicine in Rwandan medical education or routine clinical care were identified.	Rwanda-specific evidence is emerging for narrative therapy and related psychosocial interventions among genocide survivors, but these findings should not be directly generalized to narrative medicine.
Implementation implications for Rwanda	Begin with modest, voluntary, trauma-responsive educational modules; include safeguards, faculty development, referral pathways, and evaluation.	Should remain within mental health services or supervised psychosocial programs; ordinary clinicians should refer rather than provide psychotherapy beyond their competence.

Narrative medicine is not without critique. Woods has argued that the medical humanities' embrace of narrative rests on a largely unexamined assumption that human beings are inherently ‘narrative selves' and cautions against treating narrative engagement as intrinsically beneficial, regardless of context (30). A further, context-specific caution concerns transferability: scholars working on medical humanities in low-resource African settings note that funding constraints, crowded curricula, and small numbers of humanities-trained faculty make direct adoption of high-income curricula difficult without substantial local adaptation (37). In Rwanda's post-genocide context, where collective trauma intersects with established communal narrative practices such as Gacaca and sociotherapy, these cautions become especially important. Rather than diminishing the value of narrative medicine, they support a cautious, locally codesigned, and empirically evaluated approach instead of assuming straightforward transferability.

### Rwanda's narrative culture and healing practices

Rwanda has a rich heritage of oral storytelling, witnessing, and communal remembrance. Such traditions have become a vital component in the healing and reconciliation processes following the genocide. Community-based sociotherapy initiatives have employed organized group storytelling to restore trust and alleviate psychological distress, with assessments recording enhancements in emotional well-being and social cohesion among participants (28, 38, 39). Rwanda's community-based post-genocide court system, Gacaca, considered genocide-related cases through public testimony and community participation. The process demonstrates that oral narrative has been socially and politically significant in post-genocide Rwanda. However, these practices should not be treated as direct precedents for clinical narrative medicine. Public testimony, legal accountability, community sociotherapy, psychotherapy, and routine clinical listening differ in purpose, power relationships, confidentiality, voluntariness, and emotional risk. In healthcare settings, narrative practices must therefore avoid pressuring patients, students, or survivors to disclose traumatic experiences. They must also protect privacy, psychological safety, cultural sensitivity, and access to referral when distress emerges. Genocide-related personal and family histories may shape how patients and healthcare professionals respond to sensitive narratives. However, no Rwanda-specific evidence was identified on how these histories influence clinical communication or participation in narrative medicine. This uncertainty reinforces the need for culturally sensitive, voluntary, and confidential approaches that avoid forced disclosure or assumptions about individuals' backgrounds.

Evidence from sociotherapy, narrative therapy, and community-based reconciliation initiatives suggests that narrative engagement contributes to collective healing, social reconnection, and meaning-making within the Rwandan context. However, its translation into formal clinical practice and medical education remains limited and insufficiently explored (28). This distinction is important. Existing Rwanda-specific evidence supports the relevance of narrative forms in community healing, sociotherapy, testimony, and psychotherapy. Still, it has not yet demonstrated that narrative medicine improves clinical communication, medical education, clinician well-being, or patient outcomes in Rwanda. The present review consequently approaches Rwanda's narrative traditions as contextual resources for careful adaptation, not as evidence of effectiveness. Rwanda-specific research also shows that narrative therapy has been used in psychosocial care (40), but this evidence concerns psychotherapy and should not be generalized to narrative medicine or health professions education. Given the small sample and qualitative design, this finding should be read as preliminary and hypothesis-generating rather than as an established effect of narrative approaches on PTSD.

Narrative medicine and narrative therapy are related but should remain professionally distinct. Narrative therapy is a psychotherapeutic intervention that requires appropriate mental health training, supervision, and referral pathways. It may involve structured work with traumatic memories, identity, family relationships, and re-authoring life stories. Narrative medicine, in contrast, is primarily an educational and clinical practice that helps ordinary clinicians listen more attentively, interpret illness stories more responsibly, communicate more clearly, and reflect on their responses to suffering. A clinician trained in narrative medicine should not attempt to provide psychotherapy unless separately and formally qualified to do so and should instead refer patients showing signs of significant distress to appropriately trained mental health professionals. In Rwanda, this boundary is especially important because genocide-related narratives may involve severe distress, grief, shame, fear, or social sensitivity. Narrative medicine training should therefore include recognizing distress, basic supportive communication, the limits of the clinician's role, and referral to mental health professionals when needed. Table 1 summarizes these distinctions and their implementation implications.

### Stress and emotional burdens for healthcare workers

Rwandan healthcare workers operate under tough conditions due to a shortage of healthcare workers, numerous patients, and scarce mental health facilities. Patients with trauma histories from their experiences of grief and loss often experience suffering, something that healthcare professionals frequently encounter. Hearing trauma narratives could lead to the development of secondary traumatic stress and emotional exhaustion, which can negatively impact the ability of healthcare professionals to provide effective care to their patients.

Studies in post-conflict contexts have documented elevated levels of psychological distress among healthcare workers and mental health professionals (41, 42). Clinicians who lack clinical narrative thinking skills, particularly the capacity for narrative care and self-reflection, are at risk of what scholars term “narrative disconnection” or “narrative foreclosure,” a state in which clinicians become unable to engage with or be moved by patient stories (15, 43). This can result in diagnostic errors, therapeutic failures, or secondary trauma for the clinician (44).

Research conducted in post-conflict environments such as Gaza indicates elevated levels of distress, workforce strain, and burnout among healthcare professionals (42). These findings highlight a broader systemic problem: health systems frequently lack sufficient mechanisms for reflection, emotional processing, and peer support essential for sustaining clinician resilience in high-volume environments.

Narrative practices may provide structured opportunities for reflection and relational connection, but evidence that they prevent burnout remains limited. Their relevance to health professions education lies more directly in helping clinicians recognize their emotional responses, remain attentive to patients' experiences, and seek appropriate support when emotionally demanding encounters affect their practice.

### Gaps in medical education

With the conceptual grounding and cultural context established for interpreting narrative medicine responsibly, this section turns to the review's central concern: how narrative competence might be incorporated into Rwandan health professions education. Rwanda is currently expanding its health workforce and reforming health professional training (4). This creates an opportunity to strengthen not only technical competence but also communication, reflection, ethical listening, and trauma-responsive care. However, published Rwanda-specific data on narrative medicine training are scarce. We did not identify empirical studies evaluating reflective writing, narrative competence curricula, clinical narrative thinking, or trauma-responsive narrative medicine modules in Rwandan medical schools. Stating this absence is essential rather than filling the gap with unsupported assumptions. Given the limited availability of direct Rwandan curriculum data, this section presents the educational argument as a contextually grounded hypothesis. Rwanda's post-genocide mental health burden, the emotional demands placed on clinicians, and the expansion of medical training suggest a need for educational approaches that help clinicians recognize suffering, communicate with dignity, and reflect on their own emotional responses. International literature on reflective writing, patient narratives, and discussion-based learning suggests potential benefits for empathy, professional identity formation, and patient-centered communication (14, 45, 46). These findings justify local exploration, but they do not replace Rwanda-specific feasibility and safety studies.

Trauma-informed care provides the principles needed to promote safety, choice, and avoidance of re-traumatization. Narrative medicine may complement this approach by training healthcare professionals to listen closely, interpret patients' illness experiences, reflect on their responses, and communicate without pressuring patients to disclose traumatic experiences. Its role is therefore educational and relational rather than psychotherapeutic. Integrating these competencies into medical training may help healthcare professionals address both the clinical and psychosocial dimensions of care, particularly in trauma-affected settings.

In trauma-affected settings, such competencies may be particularly important, as they help healthcare professionals understand the unique experiences and needs of their patients who have faced significant adversity (31). Educational case discussions could help learners practice attentive listening and recognize possible distress without treating narrative activities as therapy. Listening carefully to patients' accounts may help clinicians understand how bodily symptoms are experienced and interpreted without automatically attributing them to psychological causes (26, 27). In trauma-affected settings, complex interactions among physical illness, psychological distress, social relationships, and past experiences of violence or loss may shape symptoms. However, clinicians should not assume that unexplained symptoms indicate an undisclosed trauma history. Biomedical assessment, patient choice, trauma-sensitive communication, and appropriate referral should remain central to care (31, 32).

Shared decision-making thinking is aided by narrative decision-making thinking, which acknowledges that patients and clinicians enter into the clinical encounter with their experiences, values, and expectations. This method is particularly applicable to situations with trauma, where previous experiences can influence patient preferences and reactions to care. Narrative patient education also supports communication by presenting medical information in culturally relevant, accessible formats, increasing comprehension and patient engagement (47). Narrative care and intervention thinking also provide a framework for sustaining therapeutic relationships over time, emphasizing continuous attentiveness to patients' evolving experiences as a basis for empathy, trust, and continuity of care. Training and workshops in narrative medicine have been associated with improvements in diagnostic attentiveness, reflective capacity, and reduced burnout in some studies. However, findings remain context-dependent, methodologically heterogeneous, and largely derived from high-income settings (48–50).

The health care system in Rwanda could explore narrative medicine through existing training and service structures, but implementation should begin modestly and safely:

1. Curriculum integration: Introduce short modules on illness narratives, reflective writing, attentive listening, and clinical narrative thinking within undergraduate and postgraduate medical education. Early modules should focus on listening, reflection, communication, recognition of relational mistrust, and professional boundaries rather than eliciting traumatic disclosure.
2. Trauma-responsive communication training: Teach students and clinicians how to recognize distress, respond without forcing disclosure, avoid re-traumatization, and refer patients to mental health professionals when needed.
3. Faculty development: Train educators to facilitate reflective discussion safely, manage emotional responses in groups, protect confidentiality, and identify learners or participants who require additional support.
4. Clinician well-being initiatives: Pilot voluntary narrative rounds or reflective writing groups for clinicians, with clear ground rules, confidentiality, non-judgmental participation, and access to psychosocial support. Clinicians should not be required to disclose personal or family genocide-related histories. Confidential support should be available when clinical encounters activate a clinician's own experiences or family history.
5. Patient-provider communication: Use narrative approaches to improve listening and shared decision-making in routine care, particularly for chronic illness, medically unexplained symptoms, and situations where illness meanings, family concerns, or social context affect care.
6. Research and evaluation: Begin with qualitative feasibility work, stakeholder consultation, curriculum co-design, acceptability studies, and trauma-safety evaluation before testing effectiveness through larger controlled studies.

The proposed pathway for integrating narrative medicine into Rwandan health professions education is summarized in Figure 1. The model is iterative, with educational, clinical, workforce, and evaluation components informing ongoing program adaptation.

**Figure 1:**
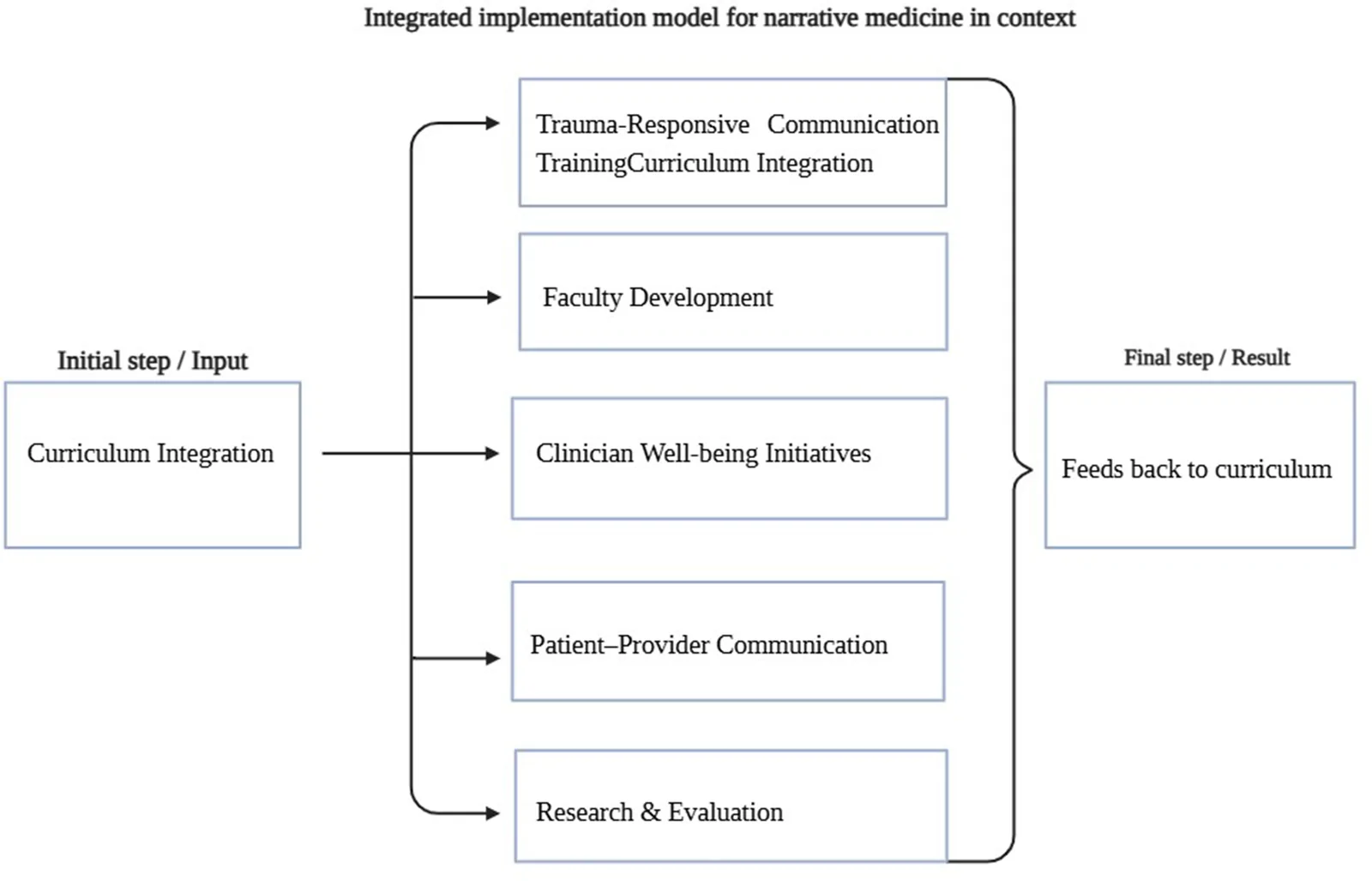
shows the model of narrative medicine implementation as an iterative system in which educational, clinical, workforce, and evaluation components are mutually reinforcing and continuously inform program adaptation.

### Ethical and safeguarding requirements for implementation

Any narrative medicine activity in Rwanda should be voluntary, carefully facilitated, and trauma-responsive. Students, clinicians, and patients should not be required to disclose personal trauma histories. Reflective writing and group discussion should have clear confidentiality rules, opt-out options, and procedures for responding to distress. Facilitators should be trained to recognize emotional overload, manage silence respectfully, avoid interpretive judgment, and maintain boundaries between education, clinical care, and psychotherapy. Where traumatic material arises, referral pathways to qualified mental health professionals should be available. Survivor narratives should never be used as teaching material without informed permission, contextual sensitivity, and protection against identification or instrumentalization.

### Implementation challenges

Several implementation challenges should be anticipated. First, medical curricula are already crowded, and faculty may have limited time or training to facilitate narrative activities, a constraint documented in other low-resource African settings (37). Second, humanities-based approaches may be perceived as less urgent than biomedical or technical training unless their educational purpose is clearly explained. Third, narrative activities can cause harm if they pressure people to disclose painful experiences, expose confidential information, or blur the boundary between education and psychotherapy (32). In Rwanda, these risks are heightened by the personal, family, social, and political sensitivity of genocide-related memories. Healthcare professionals are also part of Rwanda's post-genocide history and may bring their personal or family experiences into clinical encounters. These experiences may include survival, bereavement, displacement, rescue, accusation, or imprisonment, as well as family or community histories associated with perpetration, contested memory, or intergenerational distress. Training should therefore help clinicians recognize and manage emotional reactions such as grief, guilt, defensiveness, identification, or avoidance without requiring them to disclose personal or family histories. Confidential supervision and peer support should be available, alongside opportunities to seek consultation, transfer care, or refer patients when an encounter becomes emotionally difficult or compromises effective care. These safeguards should protect healthcare professionals, patients, and learners. Implementation should therefore begin with low-intensity, voluntary, skills-focused activities; should avoid mandatory trauma disclosure; and should include facilitator training, referral pathways, and ongoing evaluation of psychological safety.

## Discussion

This review suggests that narrative medicine may have particular value in Rwanda as an educational approach for strengthening how health professionals listen, reflect, interpret patients' experiences, and communicate in emotionally demanding encounters. Its contribution is not simply to encourage empathy but to develop a more disciplined form of narrative attention that considers emotion, meaning, silence, context, and the clinician's own responses. In this sense, narrative medicine may complement existing efforts in health professions education by strengthening social and relational competencies alongside clinical knowledge and technical skills.

These competencies overlap with aspects of emotional intelligence, particularly emotional awareness, perspective-taking, reflective self-awareness, and empathic communication, but the concepts should remain distinct. Emotional intelligence concerns broader abilities to perceive, understand, and regulate emotion, whereas narrative competence focuses on how clinicians attend to, interpret, and respond ethically to patients' stories and lived experiences. Narrative medicine should therefore not be presented as emotional-intelligence training itself. Rather, it offers a practical educational setting in which clinicians can practice social-emotional and interpersonal competencies through close listening, reflective writing, discussion, and engagement with illness narratives.

This distinction is especially important in Rwanda's post-genocide context. Narrative medicine should not be expected to treat trauma, reduce PTSD, or replace mental health care. Its more defensible role is educational and relational: preparing learners and clinicians to engage sensitively with difficult narratives, recognize emotional responses in themselves and others, maintain professional boundaries, and refer patients when needs extend beyond their competence. The international evidence on empathy, reflection, communication, and professional formation provides a rationale for local exploration, but Rwanda-specific effectiveness remains untested. Any integration should therefore begin with culturally adapted, voluntary, and carefully evaluated educational approaches rather than assuming that benefits observed elsewhere will transfer directly.

### Limitations

This review has four main limitations. First, Rwanda-specific empirical evidence on narrative medicine in medical education and clinical practice is currently very limited. The manuscript therefore relies on global narrative medicine literature and Rwanda-specific literature from related areas such as sociotherapy, testimony, reconciliation practices, trauma care, and narrative therapy. Second, the search and synthesis process was interpretive rather than systematic, and we did not conduct a formal risk-of-bias assessment or meta-analysis. Third, most evidence on narrative medicine education comes from high-income settings, which limits direct transferability to Rwanda. Fourth, the review does not evaluate an implemented curriculum or clinical intervention; it proposes a framework for future co-design and study.

### Future research

Future research in Rwanda should proceed stepwise. The first stage should include stakeholder consultation with medical students, clinicians, educators, mental health professionals, patients, survivor representatives, and ethics experts. The second stage should involve curriculum co-design and qualitative feasibility work to determine what forms of reflective writing, narrative discussion, and communication training are acceptable and safe. The third stage should pilot brief narrative medicine modules and evaluate acceptability, emotional safety, facilitator readiness, and perceived educational value. Only after feasibility and safeguarding have been established should larger studies examine effects on narrative competence, social and relational competencies, communication, professional formation, and clinician well-being. If emotional intelligence is evaluated, it should be measured as a distinct construct rather than inferred from changes in narrative competence. Randomized trials may eventually be useful, but they should not be the starting point in a trauma-sensitive educational context.

## Conclusion

Narrative medicine may offer a context-sensitive approach to strengthening narrative and relational competencies in Rwandan health professions education. Through attentive listening, perspective-taking, reflection, and empathic communication, it may help learners prepare for emotionally demanding clinical encounters. These capacities overlap with aspects of emotional intelligence but remain conceptually distinct because narrative competence focuses specifically on interpreting and responding ethically to patients' stories, meanings, and contexts. The clinical narrative thinking framework may provide a practical scaffold for developing these skills, but narrative medicine should remain an educational and relational practice rather than psychotherapy or trauma treatment. Its integration in Rwanda should therefore begin with locally co-designed, ethically safeguarded, and empirically evaluated educational initiatives.
